# Interventions to improve pneumococcal vaccination coverage: A systematic review and meta-analysis

**DOI:** 10.1016/j.imj.2026.100238

**Published:** 2026-01-20

**Authors:** Bogdan Cireașă, Géraldine Leguelinel-Blache, Bob-Valéry Occéan, Chris Serrand, Jean-Marie Kinowski, Florent Dubois

**Affiliations:** a Department of Pharmacy, Nimes University Hospital, University of Montpellier, Nîmes 30900, France; b Desbrest Institute of Epidemiology and Public Health, University of Montpellier, INSERM, Montpellier 34090, France; c Department of Law and Health Economics, University of Montpellier, Montpellier 34090, France; d Department of Biostatistics, Epidemiology, Public Health and Methodological Innovation, Nimes University Hospital, Nîmes 30900, France

**Keywords:** Pneumococcal, Vaccination coverage, At-risk patient, Intervention, Pharmacist

## Abstract

**Background:**

*Streptococcus pneumoniae* is a widely spread human pathogen that can cause infections as severe as meningitis. The pneumococcal vaccination is the optimal approach to prevent morbidity and mortality, but the vaccination coverage is low despite several attempts to improve it. This meta-analysis assesses the impact of the interventions to increase vaccination coverage.

**Methods:**

A meta-analytic review and a mixed-effects meta-regression on articles between 2013 and 2023 from PubMed, Web of Science and ScienceDirect that assessed the impact of interventions on pneumococcal vaccination coverage was performed. We included randomized controlled trials (RCTs) and comparative non-RCTs, and excluded reviews, non-comparative or retrospective studies, as well as the ones for which the full text was not available or did not present raw data on vaccination. A subgroup analysis was performed by study design, intervention type, facilitator and population characteristics.

**Results:**

16 articles were included out of 349 across three databases. Ten were RCTs and six were non-RCTs. Nine were from the USA, three from France and one from China, Zimbabwe, Singapore and Turkey respectively. Seven studies had physicians and four had pharmacists as facilitators. Most described direct interventions such as education and reminders. Ten focused on at-risk patients. The pooled relative risk (RR) was (1.06 [1.00, 1.13], *p* = 0.04), with an *I*^2^ of 17.05%. RCTs reported significantly lower effect sizes compared to non-RCTs (−0.69), *p* = 0.02. Indirect interventions showed lower effect sizes (−0.45, *p* = 0.06). Pharmacist-led interventions showed a positive, almost significant effect (0.43, *p* = 0.07).

**Conclusion:**

This meta-analysis offers valuable insights into the factors that influence pneumococcal vaccination coverage and serves as a guide for the development of more effective and targeted strategies.

## Highlights

• Systematic review and meta-analysis of 16 studies on pneumococcal vaccination.
• Interventions modestly increased vaccination coverage (RR = 1.06, *p* = 0.04).
• RCTs showed lower effect sizes compared to non-RCTs.
• Direct interventions outperformed indirect, healthcare professional–focused ones.
• Pharmacist-led approaches showed promising effects on vaccination uptake.

## 1 Introduction

*Streptococcus pneumoniae* (pneumococcus) is a widely spread bacterium that can infect both children and elders and is responsible for numerous diseases, from mild infections to severe, invasive ones.^1^ It is a human specific pathogen that, at first, colonizes the upper airways and leads to inflammation (e.g. pharyngitis, sinusitis) but can also ascend to the middle ear causing otitis, descend to the lower respiratory tract causing a mild pneumonia or even a complicated one. In some cases, the infection with pneumococcus can become as severe as sepsis or even meningitis.^2^

This represents a real public health issue worldwide. Especially in the case of at-risk patients (e.g. immunocompromised patients, elders), which are predisposed to have a severe form of this infection.^3^ The optimal approach, both healthcare and economic wise is through pneumococcal vaccination, which can prevent morbidity, mortality and increase the quality of life for the patients. Even with this in mind, the vaccination coverage is still low especially among at-risk patients.^4^, ^5^, ^6^, ^7^, ^8^, ^9^ Despite several attempts to increase the coverage (e.g. vaccination being covered by insurance, education) the number of people who accepted the vaccine remains extremely low in France.^10^ The unmet vaccination rates despite the provider-oriented interventions potentially indicates that a direct or mixed approach is necessary. As such, a next promising step can be raising the awareness among patients through population specific methods, involving the healthcare practitioners.^11^^,^^12^

This review proposes to assess the impact of the interventions on the pneumococcal vaccination rate and help classify by their clinical efficiency.

## 2 Methods

### 2.1 Study design

We conducted a meta-analysis to evaluate the impact of diverse interventions on pneumococcal vaccination coverage. This protocol has been registered on PROSPERO (reference number CRD42024539387).

### 2.2 Search strategy

We performed a systematic literature search using PubMed, ScienceDirect and Web of Science databases. The detailed search equations using relevant keywords are presented in Supplementary Table S1. Articles were screened by two independent authors (BC, FD) first by title and abstract and then by full text. All discrepancies were solved by introducing a third reviewer (GLB).

### 2.3 Eligibility criteria

We included articles published from 2013 to 2023, that assessed the impact of an intervention on pneumococcal vaccination coverage. We focused on randomized or non-randomized comparative studies in order to clearly capture the impact of the interventions. We excluded reviews, non-comparative and retrospective studies. We also excluded the studies for which the full text was not available and the studies that did not present raw data about the vaccination coverage.

### 2.4 Data extraction

We extracted a full record of the screened articles and constructed a database using the Microsoft Excel software (Microsoft corp., WA, USA). The extracted data included: title and first author, year of publication, country, establishment, health professionals involved, interventions, population, special groups of patients, total number of patients and total number of vaccinated patients in the two groups (control and intervention).

### 2.5 Quality assessment

The ROB2 Tool^13^ was used for RCTs. A funnel plot was used in order to assess the publication bias. Egger's test was employed to analyze the asymmetry of the funnel plot. The evaluation was conducted independently by two reviewers (BC, FD) to strengthen the outcome. Discrepancies were solved by consensus.

### 2.6 Statistical analysis

Effect sizes were calculated as relative risk (RR). The global effect was calculated by a random effects model meta-analysis and a mixed-effects meta-regression model was employed to assess the influence of moderators such as study design, population characteristics, intervention type and facilitator. To account for multiple moderator comparisons, the Benjamini-Hochberg (BH) (false directory rate [FDR] = 0.05) corrections were applied. Heterogeneity was quantified using the *I*^2^ parameter. A value of < 25% was considered low heterogeneity.^14^ Subgroup analyses were performed by study design, facilitator, type of intervention, and at-risk patients groups. In order to harmonize the intervention types, they were classified in direct (patient-oriented) or indirect (facilitator-oriented). Patients having chronic diseases (e.g. cardiovascular, respiratory, diabetes), immunocompromised, elders (≥ 65 years) or infants and young children (≤ 5 years), were considered at-risk.^15^

All statistical analysis was performed using the R Statistical Software (version 2024.09.1 + 394; R Core Team 2021, Vienna, Austria) and the “metafor” package.

### 2.7 Ethical considerations

This study is a secondary analysis of already published data and does not require ethical approval. All included studies have obtained necessary ethical approval from their respective institutions. Reporting was made in accordance with the Preferred Reporting Items for Systematic Reviews and Meta-Analyses (PRISMA) guidelines.^16^ The complete PRISMA checklist can be found in Supplementary Materials.

## 3 Results

### 3.1 Study selection and characteristics

**Fig. 1:**
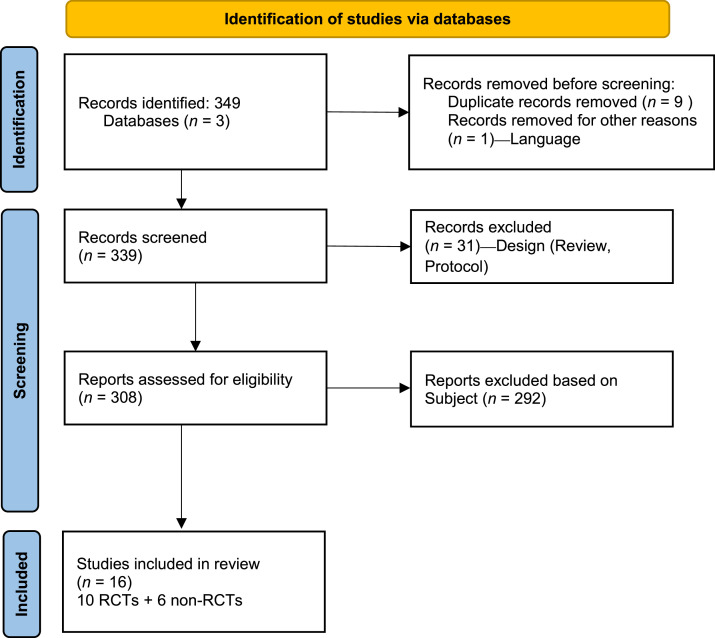
PRISMA 2020 flow diagram for new systematic reviews which included searches of databases and registers only.

We analyzed a total of 349 articles across the three databases. After removing duplicates and title and abstract as well as full-text screening, we included 16 articles in our analysis (Fig. 1). Ranging from 2014 to 2023, ten were RCTs^17^, ^18^, ^19^, ^20^, ^21^, ^22^, ^23^, ^24^, ^25^, ^26^ and six were non-RCTs.^3^^,^^27^, ^28^, ^29^, ^30^, ^31^ Nine were performed in the USA^17^, ^18^, ^19^, ^20^^,^^25^^,^^27^^,^^28^^,^^30^^,^^31^, three in the French Republic^3^^,^^21^^,^^29^ and one in China,^22^ the Republic of Zimbabwe,^23^ Republic of Singapore^28^ and Republic of Türkiye,^26^ respectively. Most studies had a medic as facilitator (*n* = 7),^21^^,^^23^^,^^25^^,^^28^^,^^28^^,^^29^^,^^31^ the other healthcare professionals being pharmacists (*n* = 4)^3^^,^^17^^,^^20^^,^^26^ and nurse (*n* = 1).^22^ Three of the studies reported a multidisciplinary approach.^27^^,^^28^^,^^30^ The interventions described were mainly addressed towards the patient (direct interventions). Type of interventions varies from educational (*n* = 6)^18^^,^^22^^,^^24^, ^25^, ^26^^,^^28^ to reminders (*n* = 2)^20^^,^^23^ and a combination of the two (*n* = 6).^3^^,^^19^^,^^21^^,^^27^^,^^30^^,^^31^ Ten of the studies focused on at-risk patient groups.^3^^,^^19^^,^^21^^,^^22^^,^^24^, ^25^, ^26^^,^^28^^,^^29^^,^^31^ Eight studies were performed in clinics,^18^^,^^19^^,^^22^, ^23^, ^24^^,^^28^^,^^30^^,^^31^ six in hospitals^3^^,^^21^^,^^25^, ^26^, ^27^^,^^29^ and two in pharmacies.^17^^,^^20^

The calculated RR for the included studies varies between 0.95 to 4.55. Detailed information about the included studies and their characteristics can be found in Table 1.

**Table 1:** Included studies' characteristics.

Reference	Title	First author	Year	Country	Design	Establishment	Facilitator	Type of intervention	At-risk patients
^3^	Impact of a clinical pharmacist’s intervention on pneumococcal vaccination in a population of at- risk hospitalized patients: The IP-VAC study.	Chiappin M.	2023	France	Non-RCT	Hospital	Pharmacist	Direct	YES
^17^	The impact of community pharmacy utilization of immunization information systems on vaccination rates: Results of a clustered randomized controlled trial.	Pamela C. Heaton	2022	USA	RCT	Pharmacy	Pharmacist	Indirect	NO
^18^	Using the 4 pillars practice transformation program to increase pneumococcal immunizations for older adults: a cluster-randomized trial.	Richard K. Zimmerman	2017	USA	RCT	Clinic	Medic	Direct	NO
^19^	Randomized controlled trial of centralized vaccine reminder/recall to improve adult vaccination rates in an accountable care organization setting.	Laura P. Hurley	2019	USA	RCT	Clinic	NM	Direct	Mixed
^20^	Effect of automated immunization registry-based telephonic interventions on adult vaccination rated in Community Pharmacies: A randomized Controlled Trial.	S. Stolpe	2019	USA	RCT	Pharmacy	Pharmacist	Indirect	NO
^21^	Effectiveness of a Multifacteted Informational-Based and Text Message Reminders on Pneumococcal and Influenza Vaccinations in Hospital Emergency Departments: A cluster-Randomized Controlled Trial.	S. Tubiana	2021	France	RCT	Hospital	Medic	Direct	YES
^22^	A nurse-delivered brief health education intervention to improve pneumococcal vaccination rate among older patients with chronic diseases: A Cluster-randomized controlled trial.	Sophia S.C. Chan	2015	China	RCT	Clinic	Nurse	Direct	YES
^23^	Effectiveness of short message services reminder on childhood immunization programme in Kadoma, Zimbabwe - a randomized controlled trial.	D. Bangure	2015	Zimbabwe	RCT	Clinic	Medic	Indirect	NO
^24^	Increasing Influenza and Penumococcal Vaccination Uptake in Seniors Using Point-of-Care Informational Interventions in Primary Care in Singapore: A Pragmatic, Cluster-Randomized Crossover trial.	Henly J. Ho	2019	Singapore	RCT	Clinic	Medic	Direct	YES
^25^	Evaluation of multi-component interventions for prevention of nosocomial pneumonia in older adults: a randomized, controlled trial.	Barbara H. Rosario	2021	USA	RCT	Hospital	Medic	Direct	YES
^26^	Impact of pharmacist-led educational intervention on pneumococcal vaccination rates in cancer patients: a randomized controlled study.	N. Ozdemir	2023	Turkey	RCT	Hospital	Pharmacist	Direct	YES
^27^	Development of a Pharmacy Technician-Driven Program to Improve Vaccination Rates at an Academic Medical Center.	Hill JD.	2017	USA	Non-RCT	Hospital	Multidisciplinary	Direct	NO
^28^	Using the 4 Pillars to increase vaccination among high-risk adults: who benefits?	Nowalk MP.	2017	USA	Non-RCT	Clinic	Multidisciplinary	Direct	YES
^29^	Effect of medical staff training on vaccination coverage in outpatients with cancer: An interventional multicenter before-and-after study.	Rivière P.	2023	France	Non-RCT	Hospital	Medic	Indirect	YES
^30^	Leveraging Interdisciplinary Teams for Pre-Visit Planning to Improve Pneumococcal Immunization Rates Among Internal Medicine Subspecialty Practices.	Shafer R.	2021	USA	Non-RCT	Clinic	Multidisciplinary	Direct	NO
^31^	A Multifaceted Intervention to Improve Influenza, Pneumococcal, and Herpes Zoster Vaccination among Patients with Rheumatoid Arthritis.	Baker DW.	2016	USA	Non-RCT	Clinic	Medic	Direct	YES

### 3.2 Pooled effects

**Fig. 2:**
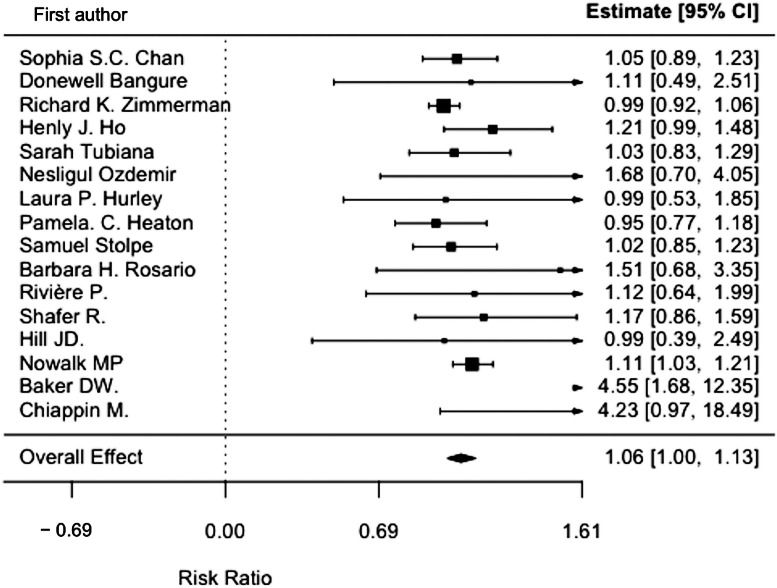
Studies' individual and overall effects.

The Random-Effects Model used returned a pooled RR of (1.06 [1.00; 1.13], *p* = 0.04). Detailed information about the overall and individual effects can be found in Fig. 2.

The Tau^2^ indicator was 0.0021 (standard error [SE] = 0.0038), while *I*^2^ was 17.05%. The model employed a restricted maximum likelihood estimator to account for residual heterogeneity.

### 3.3 Moderators effects

The overall test of moderators returned a value of 12.63, *p* = 0.12. However, individual moderators showed notable effects. RCTs reported significantly lower effect sizes compared to non-RCTs (−0.69, *p* = 0.02, 95% CI: [−1.26, −0.11]). The presence or absence of at-risk patients did not significantly influence the effect size (*p* > 0.67).

**Fig. 3:**
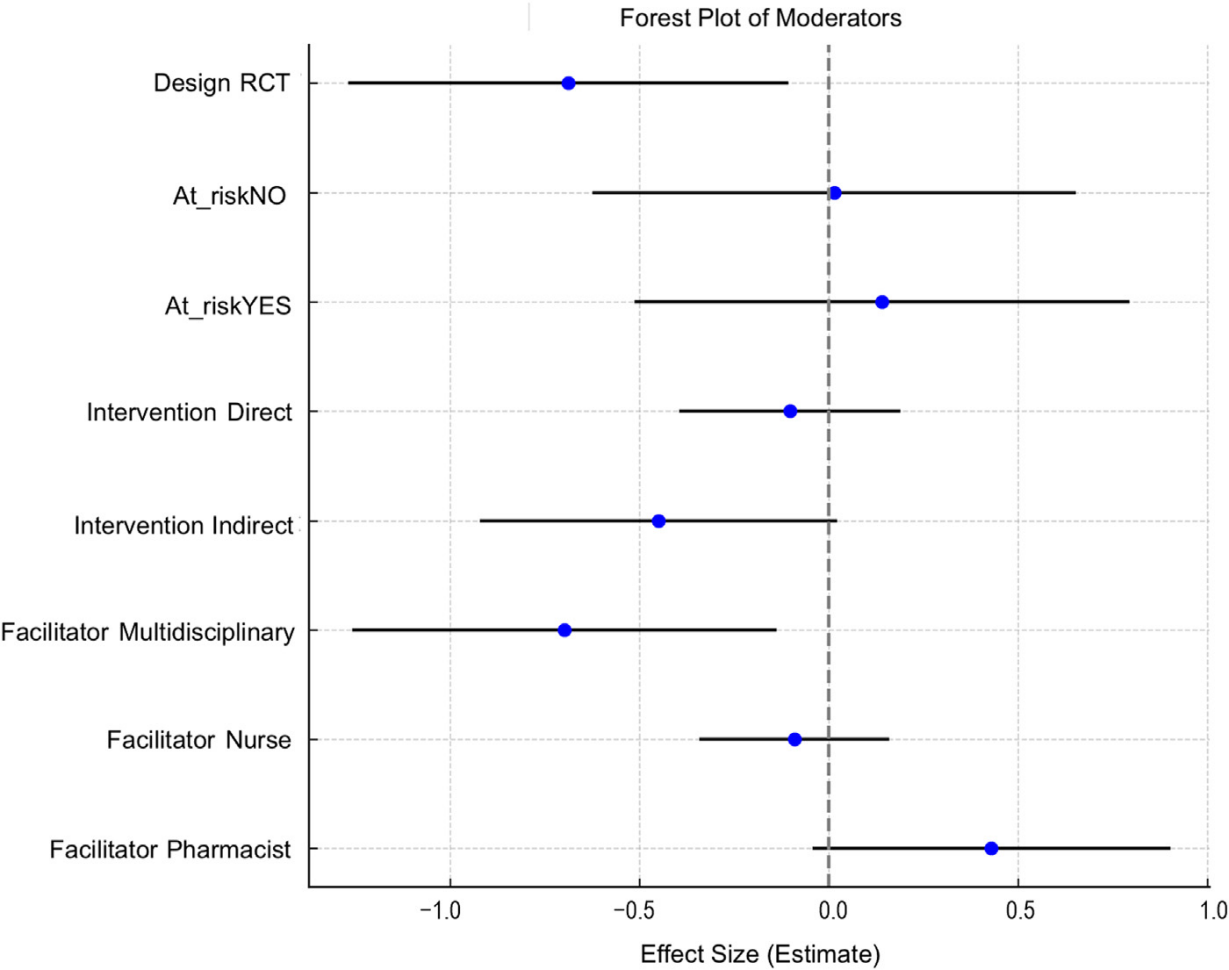
Moderators' effects.

Direct interventions had no significant impact, whereas indirect interventions showed lower effect sizes (−0.45, *p* = 0.06, 95% CI: [−0.92, 0.02]). Nurse facilitated interventions had no significant impact (*p* = 0.47), multidisciplinary teams were associated with significantly lower effect sizes (−0.70, *p* = 0.01, 95% CI: [−1.25, −0.14]), while pharmacist-led interventions showed a positive and non-significant effect (0.43, *p* = 0.07, 95% CI: [−0.04, 0.90]). These data are presented in Fig. 3. Under the BH false discovery rate, none of the moderator remained statistically significant, although effects for study design (adjusted to *p* = 0.06) and multidisciplinary teams (adjusted to *p* = 0.06) approached significance.

### 3.4 Bias analysis

**Fig. 4:**
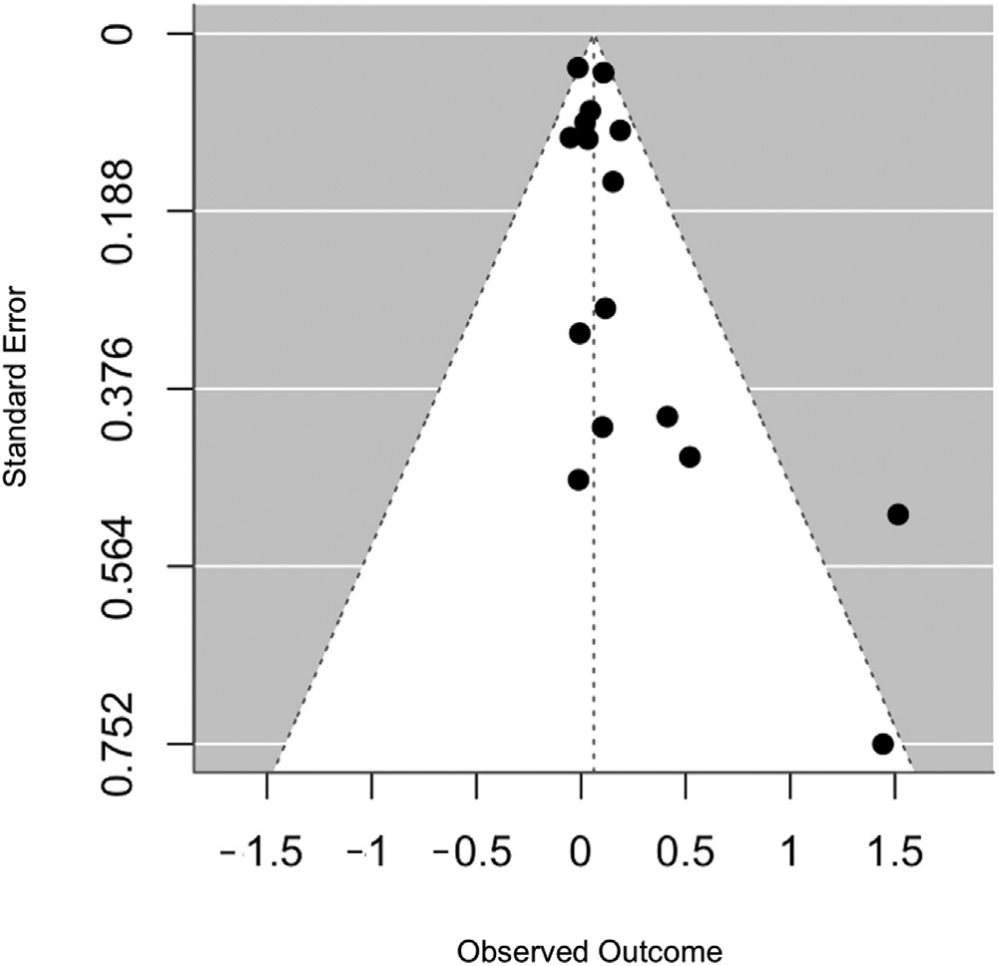
Funnel plot.

The funnel plot (Fig. 4) presented a slight asymmetry with two studies reporting overall larger effects and higher standard deviations. Egger's test returned a significant *Z* value of 2.39 (*p* = 0.02), supporting the evidence of asymmetry in the plot. We attribute this fact due to the smaller studies showing bigger effects.

**Fig. 5:**
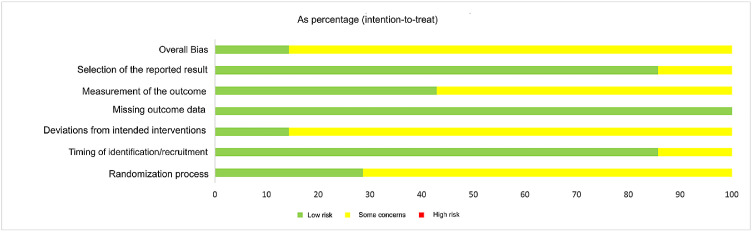
Publication bias score.

The ROB2 tool returned low overall publication bias among the RCTs. The detailed scores can be found in Fig. 5.

## 4 Discussion

This meta-analysis assessed the impact of various interventions on vaccination coverage, considering the study design, population characteristics, intervention type and facilitator. The findings characterize the interventions' effectiveness and offer potential prospects for further research.

The pooled effect revealed a modest but statistically significant overall effect of the interventions on pneumococcal vaccination coverage. This finding is consistent with the literature,^32^ both outcome-wise and regarding the necessity of more structured interventions in order to maximize the effects and their quantifiability.

The results highlight a diverse range of interventions to improve vaccination coverage across different populations and settings. The predominance of studies conducted in the USA (56.25%) suggests a potential geographical bias, with fewer studies from low- or middle-income countries. This may introduce an important issue, with overlooking the vaccination status and the potential lack of necessary interventions on one hand, as well as the lack of culturally specific approaches on the other. Unfortunately, this complex issue requires specific attention from the international entities involved in supporting the healthcare and research development in these areas. A 2016 study confirms the need of international stakeholders to apply more inclusive policies to improve research equity in these geographical areas.^33^

The majority of the interventions target the patients directly (75%), through educational strategies (37.5%). The most efficient intervention was direct and included sending educational letters to patients.^31^ On the other hand, our study reports a negative almost significant effect of the indirect approaches (i.e. targeting healthcare professionals). Moreover, the study with the lowest RR [0.95] reported an indirect intervention.^17^ On the other hand, the top four RRs were associated with direct interventions,^3^^,^^25^^,^^26^^,^^31^ two of them being > 4,^3^^,^^31^ meaning a substantial difference in the number of vaccinated patients after the intervention. Another meta-analysis focused on irritative bowel syndrome and rheumatoid arthritis patients reported that both types of interventions (direct and indirect) had a positive effect on pneumococcal vaccination uptake (RR: 0.05; 95% CI: [0.02, 0.06]).^34^ These differences can be attributed to the fact that this study evaluated an intervention on a targeted population, potentially more adherent to both direct or indirect intervention.

As expected, the physician was the most frequent facilitator (43.75%), followed by pharmacists (25%), highlighting the importance of these two healthcare professionals in promoting pneumococcal vaccination. Even if the presence of pharmacists does not have a significant effect in our analysis, the fact that they are present in a quarter of the studies underscores their involvement in the process of improving pneumococcal vaccination. Pharmacist-led interventions demonstrated a positive, albeit almost significant effect suggesting a potential role for pharmacists in enhancing vaccination coverage. These results are confirmed by another study performed on the effectiveness of the pharmacist as immunizer (RR: 1.14; 95% CI: [1.12, 1.5]) both comparing it to the usual care or without pharmacist involvement.^35^ One of the other reasons that can support this fact is the growing possibility for the pharmacist to be both facilitator and immunizer. Another meta-analysis confirms the positive impact of the pharmacist as facilitator, immunizer and both, improving the overall immunization uptake and frequency.^36^ Only a small proportion of studies assess multidisciplinary interventions (18.75%) and multidisciplinary teams were associated with significantly lower effect sizes. This can possibly be explained by the challenges in structuring interventions by coordinating the contribution of different professionals in the care pathway. Nevertheless, it would be interesting to further explore this approach, especially regarding the collaboration between physicians and clinical pharmacists.

Nurse-led interventions, however, did not show a significant impact (*p* = 0.47), which could be attributed to the variability in nursing roles and responsibilities across different healthcare settings.

Moreover, more than half of the studies focused on at-risk patient groups, underlining the specific needs of these categories, especially given the fact that this category is increasing.^10^

The included studies were homogenous in their effect sizes (*I*^2^ = 17.05%), suggesting limited variation attributable to differences in study design, populations or intervention types. This is further supported by the low (0.0021) Tau^2^ value, reinforcing the robustness of our analysis.

A notable finding was the smaller effect sizes observed in RCTs compared to non-RCTs. This can be explained by the strict methodological controls that result in more conservative results. On the other hand, non-RCTs such as comparative observational studies, may report larger effect sizes because they are more susceptible to confounders and selection bias. Furthermore, while RCTs are crucial for establishing causality with minimal bias, non-RCTs play a vital role in enhancing the external validity and applicability of research findings. By combining evidence from both study designs, we can gain a more comprehensive understanding of the effects.^37^

Indirect interventions exhibited a trend toward diminished effect sizes. Even though not statistically significant, this suggests that direct patient engagement through education or reminders may be more effective than interventions oriented towards healthcare professionals alone. A 2023 study suggests that a combination of clinician- and patients- targeted interventions is effective in increasing vaccination uptake.^38^ To account for multiple moderator comparisons, the BH corrections were applied, rendering all moderators non-significant, with the effects for study design and multidisciplinary teams approaching significance. These findings suggest that while certain moderators show potential trends, the evidence does not remain robust after correction for multiple testing, likely due to the conservative nature of these adjustments and the moderate number of comparisons reducing statistical power.

This study presents some limitations. The temporal horizon was ten years, meaning only the latest literature was taken into consideration. While this may present certain advantages, the literature search was not exhaustive. On the other hand, even though not fully accounted for, the time trends biases can be limited by the restricted period in which the articles were published. The results generated by this analysis may not be applicable to all populations, mostly due to differences in healthcare systems, but social and cultural factors as well. Given the fact that the majority of studies included in the analysis are from the USA, certain adjustments may be necessary in order to properly extrapolate them to other parts of the world. Even though the effect measure used (RR) is appropriate for this kind of analysis, some transformations and calculations were necessary in order to ensure uniformity to the data. This may introduce some inaccuracies.

Finally, the findings of this analysis can serve as a lead to stakeholders for designing efficient interventions to improve pneumococcal vaccination coverage. Moreover, we highlighted gaps in the literature, such as underrepresented populations, that future studies could address. Also, improving methodological approaches and standardizing protocols may be a solid lead in developing strong effect estimates.

## 5 Conclusion

This meta-analysis found that interventions aimed at improving pneumococcal vaccination coverage had a modest but statistically significant overall effect, with direct, patient-focused strategies—especially educational ones—proving most effective. Physicians and pharmacists emerged as key facilitators, while pharmacist-led approaches showed promising results. However, geographical and population biases persist, as most studies originated from high-income countries and focused on at-risk groups. Future research should emphasize standardized methodologies, broader geographic representation, and multidisciplinary approaches.

## CRediT authorship contribution statement

**Bogdan Cireașă:** Writing – review & editing, Writing – original draft, Software, Methodology, Investigation, Formal analysis, Data curation, Conceptualization. **Géraldine Leguelinel-Blache:** Writing – review & editing, Validation, Supervision, Resources, Project administration, Investigation, Conceptualization. **Bob-Valéry Occéan:** Writing – review & editing, Validation, Supervision, Software, Conceptualization. **Chris Serrand:** Writing – review & editing, Supervision, Methodology, Conceptualization. **Jean-Marie Kinowski:** Writing – review & editing, Validation, Project administration, Conceptualization. **Florent Dubois:** Writing – review & editing, Validation, Project administration, Methodology, Investigation, Formal analysis, Conceptualization.

## Informed consent

Not applicable.

## Organ donation

Not applicable.

## Ethical statement

Not applicable.

## Data availability statement

The data utilized in this analysis can be obtained from the corresponding author upon reasonable request.

## Animal treatment

Not applicable.

## Generative AI

Not applicable.

## Funding

This work has not received any funding.

## Declaration of competing interest

The authors declare that they have no known competing financial interests or personal relationships that could have appeared to influence the work reported in this paper.

## Appendix Supplementary materials

Image, application 1

Image, application 2
