# Supplementary material for: Interventions to improve pneumococcal vaccination coverage: A systematic review and meta-analysis
Source: Infect Med (Beijing). 2026 Jan 20;5(1):100238. doi: 10.1016/j.imj.2026.100238 (PMC12907082; doi:10.1016/j.imj.2026.100238)
Supplement: Supplementary file 1 [file mmc1.docx]

**Table S1. Research equations**

| PubMed | ScienceDirect | Web of Science |
| --- | --- | --- |
| ((pneumococcal[Title/Abstract] OR pneumococcus[Title/Abstract]) AND ((vaccin* uptake[Title/Abstract]) OR (vaccin* coverage[Title/Abstract]) OR (vaccin* rate[Title/Abstract]) OR (vaccin* delivery[Title/Abstract]))) AND (intervention[Title/Abstract]) | Title, Abstract, Keywords: (pneumococcal OR pneumococcus) AND ((vaccine OR vaccination) AND (rate OR coverage)) AND patients | (((AB=(pneumococc*)) AND TI=(vaccin*)) AND TI=(rate OR coverage OR uptake)) AND TI=(intervention) and 2023 or 2022 or 2021 or 2020or 2019 or 2018 or 2015(Publication Years) |
